# Medical Items and News of Interest to the Physician

**Published:** 1882-01

**Authors:** 


### Medical Items and News,

### For the Use of Physicians.

CULLED FROM THE STANDARD MEDICAL JOURNALS OF THE WORLD.

NOTE.—These formulæ are intended only for the regular practitioner of medicine, and should be employed only by him, or under his immediate supervision.

**Eczema.**

℞

Ung. picis, ℥ i.

Zinci oxidi, ʒ ii.

Ung. aq. Rosæ, ℥ iii.

[*Dr. Bulkley in Western Med. Reporter.*

**Covering the Taste of Bromides.**

According to the *Canadian Medical Journal*, this may be accomplished by giving three times as much simple syrup as there may be of the bromide. The result is said to be an agreeable, nutty flavor, not unlike that of cocoanut-milk.

**Danger of Ergotin.**

At a recent meeting of the Academy of Sciences M. Boissarie read an article on the inconveniences and dangers of ergotin. When given in continuous doses for a considerable period even when these are small, ergotin accumulates in the economy, and may suddenly give rise to ergotism of a severe type. He gave also the history of a case of spontaneous gangrene of the lung from ergotism.

**Mammitis.**

Dr. J. D. Smith heartily agrees with Dr. Corson in the application of ice to an inflamed or suppurating mammary gland, but claims that in many localities ice cannot be had, and gives the following as a substitute:

℞
Glycerine, ℥ i.
Fluid ext. belladonna, ℥ j.
Water, f. ℥ j.
Iod. potas, ʒ j.

**Burns.**

Dr. Shrady, of New York city, recommends that burns be treated by applying a paste composed of three ounces of gum arabic, one ounce of gum tragacanth, one pint of carbolized water (one part to sixty), and two ounces of molasses. The paste is to be applied with a brush, renewed at intervals, and is stated to be a successful method. Four applications are usually sufficient, the granulating surfaces being treated with simple cerate or the oxide of zinc ointment, as indicated.

**Cholera Infantum.**

℞

Tinct. opii, gtt. ij.

Spts. ammon. aromat, ʒ ss. to j.

Bismuth subnitrat, ʒ ij.

Syr. Simplic, ʒ ss.

Mistur. cretæ, ℥ iss. M.

Sig. One teaspoonful every two or three hours to a child of eight to twelve moths, until vomiting and diarrhœa are controlled.—*Lewis Smith, in Medical Summary.*

**Anemia of Chlorosis.**

℞

Ferri vini amari, ℥ vijss.

Tinct. nucis vomicæ, ʒ iv.

Liq. potass. arsenit, ʒ ij. M.

Sig. A desert spoonful in a glassful of water just after each meal.—*Professor T. Gailard Thomas, M. D.*

In addition to this Dr. Thomas (regarding the indications as to remove the cause, cure the neurosis and repair the damages), advises general tonic treatment and the observance of good hygiene.—*Medical Summary.*

**Gonorrhœa.**

No. 1. ℞
Liq. ferri subsulphatis gtt. xv
Aquæ font q. s. ℥ jv

No. 2. ℞

Hydrastin muriatis ℈ j

Glycerinæ puræ f. ℥ ss

Aquæ font q. s. ft. ℥ jv.

Directions.—Wash out urethra well with warm water then inject formula No. 1. Six hours after use No. 2 by injection. Four days is all I ask for cure.

This treatment has never failed where I have given it.—*Penrose Jones, in Therapeutic Gazette.*

**Infantile Convulsions.**

℞
Olei succini rectificati.
Tincturæ opii, aa ℥ ss.
Olei oliviæ.
Spiritus vini gallici aa ℥ ij.

Ft. lotio. Rub along the spine.

—*Dr. Jos. Parrish.*

The above will relieve infantile convulsions, and is also an excellent application to relieve the spasms of whooping-cough. Care should be taken to wash the skin with warm water and soap before rubbing in the lotion, to promote absorption.—*Medical Summary.*

**Simple Remedy for Burns.**

Common whiting mixed with water to the consistency of a thick cream, spread on linen, forms an excellent local application to burns and scalds. The whole burnt surface should be covered, thus excluding the action of the air. The ease it affords is instantaneous, and it only requires to be kept moist by occasionally sprinkling of cold water.—*Medical Summary.*

---

**Treatment of Hay Fever.**

Dr. Hermann Hager, who has observed a case of catarrh, with subsequent asthmatic trouble and loss of appetite, which closely resembles the hay-fever of England and the United States, thought of trying his catarrh pills prepared after the following manner:

℞
Quinidiæ sulphatis, 10.0 gm.
Tragacanthæ, 4.0 gm.
Althæa rad., 1.0 gm.
Gentian, rad., 8.0 gm.
Glycerin, 7.0 gm.
Acid. hydrochloric, 7.0 gm.

M. Make 200 pills. Take 3 every two hours.

---

**Alcoholism.**

The following mixture is in use in the Albany Hospital for the treatment of the effects of acute alcoholism, to relieve nervous excitement and insomnia:

℞

Tr. opii. deod,

Ext. hyoscyam fld., āā ʒ i.

Chloral hydrat,

Pot. bromidi, āā ʒ i.

Tr. capsici, ʒ ss.

Tr. aconiti rad, ♏ v.

Aq. menthæ pip., *ad* ℥ iv. M.

Sig —Two tablespoonfuls and repeat in four hours if sleep is not produced.—*Canada Lancet.*

---

**Calomel in Small Doses.**

A lady, aged seventy-five, for months had been sallow; frequent vomitings, with distress in right hypochondrium; stools light in color. In April last became much worse; vomited everything; increase of discomfort in right side, found liver so much enlarged that lower border reached nearly to ant. sup. spinous process. Counsel advised nitro-muriatic acid dilute. This increased distress and was refused after first trial. It looked like organic disease that was marching rapidly towards a fatal ending. Next ordered calomel and sugar of milk, one part to five, well rubbed together. One grain of the mixture was placed on the tongue every second hour until ten grains were taken. This was followed by stools darker in color, and by most marked relief. After two days' suspension the same course was repeated. The pain and distress, the vomiting and yellow skin rapidly disappeared. She left her bed in a few days and has remained well since.

In this case would any other known remedy have answered the purpose of a mercurial?—*Wm. T. Plant, M. D., Medical Summary.*

---

**Gonorrhœal Ophthalmia.**

A Dr. Doe used a 20 per cent. solution of benzoate of sodium and a 10 per cent. solution of tannin in the treatment of gonorrhœal ophthalmia with the result of recovery in a "few" days without resulting opacity. As, however, he employed also ice-compresses constantly during the treatment, and as the latter is one of the most powerful means for relieving intense ophthalmia, it is still to be doubted whether his patients might not have done just as well if he had omitted the benzoate altogether.

---

**Tasteless Cod-liver Oil.**

Dr. Peuteves, in *La France Medicale*, recommends, in order to render cod-liver oil tasteless, to mix a tablespoonful of it intimately with the yolk of an egg, add a few drops of essence of peppermint, and a half tumbler of sugared water, so as to obtain a *lait du poule.* By this means, the taste and characteristic odor of the oil are entirely covered, and the patients take it without the slightest repugnance. Besides, the oil being thus rendered miscible as the water in all its proportions, is in as complete state of emulsion as the fats at the moments they penetrate the chyle vessels, consequently absorption is better assured.

---

**Medicinal Use of Indian Hemp.**

Dr. Michel, (Montpellier Med.; Bull. Gen. de Therap., p. 380), again calls attention to the value of Indian Hemp, particularly in the treatment of uterine affections. He proposes the following formula in metorrhagia:

℞
Tincturæ cannabis indicæ, ʒss.
Syrupi simplicis, f ℥ j.
Aquæ ad f ℥ viij.

M. Sig. A teaspoonful every five or six hours.

His experience leads to the following conclusions. 1. The action of Indian hemp is double,

—excitant in small doses, in larger ones sedative and even hypnotic. 2. Of use in most nervous affections, it is particularly valuable in chorea, tetanus, certain cases of mental alienation, delirium tremens, and neuralgia. 3. The muscular tissue of the uterus is particularly sensitive to its influence; metrorrhagia is stopped by it, and the uterine contractions so increased that it might be subsituted for ergot.—*Ph. Med. Times.*

---

**Podophyllin.**

All who have been accustomed to prescribe podophyllin in pills will agree as to the impossibility of preventing occasional disastrous effects. But this is the fault of the form of administration—not of the drug. From a very long and extensive experience, I can confidently affirm that none of the accidents and inconveniences which so commonly attend the administration of podophyllin ever arise when the drug is prescribed according to my method. On the contrary, it is one of the most satisfactory and reliable of our medicines. The formula given is:

℞
Podophylli resinæ, gr. ij.
Essentiæ zingiberis, ʒ ij.
Spiritus vini recti, q. s. ad ℥ ij.
Fiant guttæ.

A teaspoonful to be taken in a wineglassful of water every night at bedtime, or every second, third, or fourth night, as required.—*Dr. Horace Dobell, in British Medical Journal.*

---

**Epilepsy, Bromides in.**

Dr. A. Hughes Bennett (*Edin. Med. Journ.*, February, 1881, p. 706,) says that the general circumstances of the individual are to be studied, his diet, hygiene, surroundings, habits, etc., if faulty, are, when practicable, to be improved. The bromides, when ordered, are to be taken without intermission. The minimum quantity for an adult to begin with is thirty grains, three times a day—the first dose half an hour before rising in the morning; the second in the middle of the day, on an empty stomach, and the third at bed-time. This is continued for a fortnight, and, if with success, is persevered with, according to circumstances, for a period varying from two to six months. If, on the other hand, the attacks are not materially diminished in frequency, the dose is immediately increased by ten grains at a time until the paroxysms are arrested. In this way as much as sixty to eighty grains thrice daily have been administered by Dr. Bennett, and, with one or two isolated exceptions, he has never found a case which resisted the treatment. He has never seen any really serious symptoms of poisoning or injury to the general health. Sometimes these amounts must be continued, but it is better to diminish to that dose, which will keep the attacks in check without seriously interfering with the patient's health. His experience with three hundred cases (of which he gives tables) has been so favorable as to lead him to believe epilepsy, of all chronic nervous diseases, is the one most amenable to treatment. Dr. Bennett's favorite prescription is as follows:

℞
Potass. bromidi.
Ammon. bromidi, aa grs. xv.
Sp. ammon. aromat, f ʒ ss.
Infus. quassiæ ad., f ℥ j.
M. Sig.—Thrice daily.—*Medical Times.*

---

**Fissured Nipples.**

Dr. Fordyce Barker recommends the following lotions:

℞

Acidi tannici, ʒ ii.

Glycerine.

Aquæ rosæ, aa ℥ ii.

In my hands it has seldom proven to be of service, except in mild cases.

℞
Plumbi nitrat., gr. viii.
Aquæ puræ, ℥ i.
M.

Dr. A. H. Smith recommends:

℞

Emplast plumbi, ʒ ii.

Ether sulph., ʒ ss.

Collod. flexil., ℥ i.

M. S.—Apply with a brush.

The following makes a moderately fair wash:

℞
Acidi tannici.
Acidi carbolici, aa gr. v.
Aquæ rosæ, ℥ i.
M.

Some practitioners dust the nipple with subnitrate of bismuth, others use borax, etc.

Various kinds of nipple shields are suggested. The simple rubber nipple is by far the best, and I rely upon its use in these cases almost to the exclusion of any medicated lotion, and I think you will always adopt this treatment if you will follow certain rules.—*Western Med. Reporter.*

**Earache.**

Dr. Morgan states that he had often promptly relieved the distressing earache of children, by filling the bowl of a common new clay pipe with cotton wool, upon which he dropped a few drops of chloroform, and inserting the stem carefully into the external canal, and adjusting his lips over the bowl, blew through the pipe, forcing the chloroform vapor upon the membrana tympani.—*National Medical Review.*

---

**Salicylic Acid in Small Pox.**

℞

Acid salicylic, ʒ j;
Spts. vin. rectificati, ℥ ss.
Mix and add:
Elix. simplici., q. s. to, ℥ vj.

For the angina of variola with the above, a gargle of xylol is prepared as follows:

Xylol, ʒ j.

Gum acacia, ʒ ij.

Aq. menth. pip, ℥ vj.

M. Ft. Emul. Sig.—Use as a gargle and mouth wash.—*Dr. Boyer.*—*Western Medical Reporter.*

---

**Ethylate of Sodium.**

In the London *Lancet* for April, 1881, American edition, Dr. B. W. Richardson strongly recommends the ethylate of sodium in the vascular form of nævus materni. These are very troublesome affections, and may be situated in parts which would make it dangerous or impossible to remove with the knife. He operated on seven children with success in every instance. The nævi were chiefly situated on the head and face. A pure solution of the ethylate was freely applied by means of a glass rod to the side of the nævus. Following the application an eschar forms, which in a short time drops off, when the ethylate is applied again till the whole is destroyed. No scar is caused by it. Water must not be used when the eschar separates, nor must the eschar be separated by any violence.

Dr. Richardson applied it in the removal of tattoo marks, nasal polypus, ozœna, lupus and other affections of the skin and mucous membranes with success.—*Pittsburg Med. Journal.*

---

**Felons.**

Dr. J. H. Stearns, of Milwaukee, writes the *Therapeutic Gazette*, concerning them, as follows:

Felons are of an erysipelatous character, and not unfrequently arise from a slight external puncture or other injury. The fact that pressure causes absorption, is a well known physiological law, and this principle should be the basis of the treatment for this most painful of afflictions.

The course that I pursue is this: the finger or part is first bathed with a solution of carbolic acid until the skin is slightly bleached, and then after wiping dry the part is varnished with collodion, covering it thick enough to form a good coat; on drying, this contracts, and produces a pressure that is temporarily very painful, and should be relieved by an anodyne. In such case the bowels are almost uniformly constipated, and a cathartic is required. If treated early this plan aborts the felon almost invariably, and if matter has already formed, it comes at once to the surface, and an incision through the skin allows it to escape. The most deceptive treatment is by poulticing; it invites suppuration, and entails weeks and perhaps months of suffering, which should be confined to only a few days. The plan here recommended has been employed in hundreds of cases with the most gratifying results.

---

**Croup.**

The use of the catheter in croup or œdema glottidis has been recommended in several quarters. Dr. J. Wilson Paton, of Rockferry, England, relates in the *British Medical Journal* a case where this method was successfully employed. A child, aged three years and ten months, was attacked with croup, following measles. The symptoms of obstruction of the larynx gradually increased in severity. Dr. Paton was at last called in at 1:30 A. M., and found the patient suffering from intense dyspnœa, quite unable to speak, and the lips and face cyanosed.

The respirations were 37 per minute; pulse 144, and very weak. It was evident that the child could not live long unless it got some relief. A No. 11 gum-elastic catheter was very easily passed through the larynx into the trachea. The child made violent struggles to expel the tube, the face becoming livid and the eyes staring. In a minute or two the struggles ceased, and inspirations, partly through the tube, and partly through the larynx, were made. Considerable blood and mucus were ejected through the tube and mouth. After a time the cyanosis and dyspnœa lessened, the child lay quiet, and was able to swallow milk. Cough continued at intervals of ten minutes, about as before. The tube was removed at the end of eleven hours. Shortly after this, symptoms of obstruction appeared again, and a No. 12 catheter was introduced, this time with very little struggle following. In a course of a few hours respiration and pulse became lower, and dyspnœa ceased. The tube was kept in for forty-eight and one-half hours, and was not inserted again. The child made a rapid recovery. Dr. Paton thinks it would certainly have died had it not been for the use of the catheter.—*Cin. Medical News.*

---

**Skin Diseases.**

There is, perhaps, no more important medicinal property belonging to Peruvian balsam than that which it exercises in a variety of diseases of the skin.

In all cutaneous affections which are characterized by itching, it is specially indicated. Perhaps it is owing to this peculiar property that much of its beneficial influence is due, the *rationale* of which is obvious.

Many old, chronic cases of psoriais and eczema, attended by intolerable itching, have been relieved by one application; a continuation of the remedy, with coadjutants, resulting in complete acesia.

It may be combined with carbolic acid, cosmoline and other medicines, when indicated.

An ulcer of twenty-five years' standing, entirely around the leg, from two inches below the ankle half way to the knee, in a patient nearly 70 years' old, rapidly healed under the following application, together with alterant and roborant medication, and local support to the enfeebled capillaries.

℞

Olei petrolei (cosmoline) ℥ iv.
Myroxylon Peruiferi, ℥ ij.
Acidi carbolici, ʒ ij.
Chloral, hydrat, gr. xxv.
Morphia acetatis, gr, xv,
Thymol, gr. x.
Bismuthi subcrab, ʒ ii.
Zinci oxidi, ʒ ii.

M. Sig.—Apply once a day.—*By J. B. Garrison, M. D., Williamette, Ark., in West. Med. Reporter.*

---

**Asthma and Insomnia.**

Dr. William Murrell, in the *British Medical Journal*, writes: There can be no question as to the value of fuming inhalations in the treatment of asthma. The ordinary nitre paper often fails, because it is not strong enough. For some time past I have been in the habit of using very thick and strong nitre papers, which may be called "nitre tablets." They contain both chlorate and nitrate of potash. Each consists of six pieces of white blotting paper, about six inches square, and they are made by dipping them into a hot saturated solution of nitre and chlorate of potash. Before the pieces are quite dry, they may be sprinkled with Friar's balsam, spirit of camphor, tincture of sumbul, or some aromatic. The nitre paper so prepared is as thick as card-board, each piece consisting of six pieces of blotting paper, closely adherent, and covered all over with crystals of saltpetre and chlorate of potash. The door and windows having been closed, the tablet is placed on a fire shovel, or piece of metal, of some kind, and folded down the middle, so as to make it like a tent or the cover of a book. When lighted at each end it burns very quickly, throwing out a flame often four or five inches long, and giving rise to dense volumes of smoke. The asthmatic patient almost immediately obtains relief, and drops off into a quiet slumber, from which he awakes refreshed. These tablets often succeed when the ordinary nitre papers do no good. They nearly always induce sleep, and I have used them with success in cases of insomnia when most of the ordinary remedies have failed. Large pastils, composed of equal parts of nitre and lycopodium, are also useful in asthma.—*Chem. and Drug.*

---

**Pneumonia.**

Dr. S. C. Cook, of Wright City, Mo., says in the last number of the *American Medical Journal* as follows: In reply to your request for pneumonia treatment, in March number of your journal, I append my treatment for an adult:

℞

Tinct. ver. vir. (Norwood's), xx gtt.
Fluid ext. ipecac, xxx gtt.
Fluid. ext. lobelia, gtt. xxv.
Aquæ, ℥ iv.

M. S.—Teaspoonful every hour. Also:

℞.

Fluid ext. lobelia seed, ʒ iv.

Lard, ℥ iv.

M. S.—Mix thoroughly and spread on cloth thinly, and apply over the chest to the extent of the dullness Avoid letting the plaster extend over the stomach, or it will produce emesis. If the pulse is weak, tinct. aconite root may be used instead of veratrum in the first prescription in the proportion of xxx gtt to water ℥ iv. Now, sir, the above treatment will positively cure any uncomplicated case of pneumonia, in from twenty-four to forty-eight hours, as I have proven in 112 cases in the last eighteen months, without a single exception or funeral. Get your medicine from a reliable house, and don't substitute, and you will cure every case.—*Medical Summary.*

**Constipation, Tonic.**

I am not pleased with my trials of cascara sagrada in habitual constipation, nor was my confrere, Dr. James B. Gage. I much prefer the following formula which allow me to recommend to you:

℞
Quiniæ sulph., ʒ ss
Piperin, gr. xv
Ext. nucis vom., gr. iv
Hydrarg. sub. mur., gr. xv.
M. Divide in pills No. 30.

Sig. One pill night and morning. I have never, in 37 years practice, known this pill to fail in relieving habitual constipation, and when given in conjunction with Eberle's old formula of iron and quinine, I have never known it to fail in relieving any case of malarial cachexia. My own tonic in the latter cases is:

℞

Ferri sulph. exsic. ʒ jss

Sacch. alb. purif., ℥ jss

Aquæ aurantii flor. ℥ iij

Quiniæ sulph., ʒ j

Acidi sulph. arom. q. s. ad. solve.

Tr. cinchonæ comp. ℥ jvss

Tr. aurantii cort. ℥ ss

M. fit. sol. filter.

Sig. A desert spoonful three times a day before meals.—*Benj. D. Lay, M. D., in Therapeutic Gazette.*

**Dyspepsia in Infants.**

Dr. Steiner recommends in his Compendium of the diseases of children the following formulæ, which he has often employed with good results in the treatment of dyspepsia in young children. Dyspepsia, the result of overloading the stomach with difficultly digestible and badly assimilated foods, is a condition which is frequent in those who are brought up by hand. The treatment of such cases requires careful attention to diet, and where there is an excessive acidity of the stomach, magnesia and bicarbon ate of soda may be employed as follows:

Sodæ bicrb. 0.20—0.59 centigrams.
Aq. destill. 80 grams.
Sprup simpl. 10 grams.
Sig. A desertspoonful every two hours.

In those cases where there is excessive alkalinity on the other hand, acids in a very dilute form are specially indicated, and of these more especially hydrochloric acid:

Acid hydrochlor. dilut. gtt. x.
Aq. destill. 70 grams.
Syrup simpl. 10 grams.
Sig. A teaspoonful every two hours.

With very young children a dose of a centigram of pepsin may be administered before each meal.

The dyspepsia of older children, due to improper diet, can sometimes be quickly cured by an emetic and strict attention to diet. Colic of a dyspeptic character, may often be cured by the employment of the following:

Sodæ bicarb. 0.50—0.80 centigrams.
Aq. fœnicul. 8o grams.
Syrup diacod. gtt. xv.
Sig. A teaspoonful every two hours.

If their is constipation this mixture may be ordered:

Hydromel 40 grams.
Aq. fænic. 40 grams.
Aq. lauroceras, gtt. xv.
Sig. A teaspoonful to be taken every half hour until the medicine acts.—*Le Progres Med.*

**Diphtheria.**

Dr. G. W. Garrison, in the Cincinnati *Lancet and Clinic*, advises the following treatment:—First twenty-four hours: Saline cathartic. Topical: tincture iodine all over the cervical region, paint it every four to six hours. Locally:

℞

Hyposulphite sodæ, ℥ i.

Aq., menth, pip., ℥ vij.

Bro. ammonium, ʒ iv. Mix.

Gargle every two hours and take one teaspoonful at same time.

Constitutional treatment:

℞
Carb. ammonia, ℥ ss.
Acid acct. dil., f ℥ i. Mix.
After effervescing add:
Tinct. ferri chloridi, ℥ i.
Glycerin pure.
Aq. Menth. pip. aa q. s.
Ft. oz. viij. (f. oz, viij).
Sig. One teaspoonful every two hours.

At the expiration of forty-eight hours we use the iron mixture every two hours, alternating with whisky and quinine, and continue the latter until our patient is perfectly restored. Topical applications are of but little consequence after two or three days. In place of the iodine some oleaginous mixture should be applied to the neck for two or three days.

---

**Chronic Intermittent Fever.**

The ordinary forms of intermittent fever I treat with a great deal of success, by the following combination, a teaspoonful of which is taken every two hours, during both the cold and hot stages, for several days successively, omitting it only during the hours of rest at night, or when there is no recurrence of the chill after the third or fourth day ef its employment. You will notice that it is a combination of the antiperiodic with a febrifuge to meet the indications of both stages:

℞

Quiniæ sulph., ℨ i.
Acid. sulph. aromat., q. s.
Elix. glycrrhiz. comp,, ℥ i.
Spt. ether. nit., ℥ j.
Spt. mindereri, ℨ vi.
Tinct. aconit., gtts. xx.
Syr. limonis, q. s. ℥ iv.

Occasionally cases of rare or more persistent form present themselves for treatment. In these cases I have met with uniformly good results from the following combination, as alterative, prescribed during the interval of attack, and up to a day or two of the expected recurrence, when the quinine mixture given above is to be renewed, and continued until after the danger is past:

℞

Tinct. iodinii.

Tinct. sanguinariœ.

Tinct. ferri chlo., aa ℥ i.

M. Sig.—From twenty to thirty drops, in water, three times daily, after meals.—*Med. Sum.*

---

**Scarlatina.**

On page 23, of the transactions of the Illinois State Medical Society, which met at Jacksonville, Ill., on the 18th of May, 1875, will be found an article read by me at said meeting, entitled, "A New Plan of Treatment of Scarlatina, and How to Prevent its Spread," from which I quote the following, viz. "Believing that the disease is communicated by or with the desquamation, I keep the skin of the patient saturated with oil, it is impossible for them to circulate through the air on account of the specific gravity of the oil."

This has proved so perfect a preventive that I have frequently known the disease to be confined to one room of a large house where more than one family with several children resided.

The oils prevent the distribution of the poison through their specific gravity, and the iodide of potash prevents taking the disease by meeting and destroying the poison in the blood I have not used carbolic acid with the oils, but have used it and the various chlorinated preparations about the room and bed.

If it be clearly proved that carbolic acid or any other agent that can be used with oils will surely destroy the life of the germs of scarlatina, we shall find fair sailing on future voyages among the rocks, snags and rapids of the meandering current of this truly to be dreaded scourge; for I am most profoundly convinced that scarlatina is never communicated by any other means than by and with the branny scales from the skin of the sick.

I am of the opinion that carbolic acid and other disinfectants should be thoroughly tested in this disease, and that iodide of potash is worth more in the treatment of this complaint than other remedies, alone or combined, that have ever been used by our profession.

The fact that I have not met one case of anasarca, or abscess where the remedy had been used from the commencement of the attack until the skin was fully restored to its healthful state, is of sufficient import, in my humble opinion, to warrant the profession giving it a fair trial.

Since 1875, I have examined the urine of a great number and variety of cases of scarlatina, and find that albumen diminishes in a fair proportion to the constitutional effects induced by the iodide; and to this I mainly attribute the immunity of my patients from dropsical effusions and other sequences.

I used carbolized cosmoline last winter and spring to allay the terrible itching attending measles, with the most pleasing results in every case.—*Dr. J. P. Walker, in Western Medical Reporter.*

---

END OF 1881.—Now pay for your medical ournals and start the New Year right.

